# Predictive value of monocyte to high-density lipoprotein cholesterol ratio and tumor markers in colorectal cancer and their relationship with clinicopathological characteristics

**DOI:** 10.1186/s12957-023-03079-6

**Published:** 2023-07-08

**Authors:** Xuan Zhang, Hongyan Qin, Xiaodan Tan, Yuncong Mo, Zhenyong Li, Guofeng Huang, Zhixiao Wei

**Affiliations:** Department of Nuclear Medicine, The First Affiliated Hospital of Guangxi Medical University, 6 Shuangyong Road, Nanning, 530021 Guangxi Zhuang Autonomous Region China

**Keywords:** Monocytes to high-density lipoprotein cholesterol ratio, Colorectal cancer, Carcinoembryonic antigen, Carbohydrate antigen 199, Tumor markers

## Abstract

**Objective:**

To evaluate the predictive value of monocyte (M) to high-density lipoprotein cholesterol (HDL-C) ratio (MHR) and tumor markers in colorectal cancer (CRC) and their correlation with clinicopathological characteristics.

**Methods:**

Hematology test data and medical records of 202 CRC patients and 201 healthy subjects were collected retrospectively. The diagnostic efficacy of MHR was evaluated using receiver operating characteristic (ROC) curves and risk factors for CRC were analyzed by multivariate logistic regression.

**Results:**

CRC patients had significantly higher M, MHR, carcinoembryonic antigen (CEA), and carbohydrate antigen 199 (CA199) levels, but significantly lower HDL-C levels than healthy controls (all *P* < 0.05). Additionally, MHR was positively correlated with tumor differentiation in CRC patients (*P* = 0.049); CEA and CA199 levels in CRC patients increased with increased stage, lymph node metastasis and tumor size ≥ 5 cm (all *P* < 0.05). Furthermore, high levels of MHR, CA199 and CEA were independent risk factors for CRC. The area under ROC curve of MHR combined with CEA and CA199 was 0.882/0.869 for the diagnosis of CRC, respectively.

**Conclusion:**

This is the first study to explore the predictive value of MHR in CRC, and its continuous increase is an independent risk factor for CRC. MHR is a promising predictor for CRC progression along with CA199 and CEA.

## Introduction

Colorectal cancer (CRC) refers to cancers that arise from the colorectal epithelium, including colon cancer and rectal cancer. According to statistics, CRC mortality in the United States ranked second among all malignancies in 2020 [1, 2]. Similarly, CRC is the most common malignancy in China [3]. Due to changes in lifestyle, dietary structure, aging population, and lack of regular physical examination in recent years, the number of CRC cases has increased annually, seriously affecting people's quality of life. The 5-year survival rate of CRC patients decreases with increased diagnosis time, late tumor stage (WHO classification), and significant symptoms [4]. Improvement in the prognosis of CRC patients largely depends on the early diagnosis of CRC, tumor size, pathological differentiation, and WHO classification of tumor. Therefore, finding hematological markers for early CRC diagnosis is vital for the survival and prognosis of patients [5, 6].

Monocyte count (M; a hematology indicator) to high-density lipoprotein cholesterol (HDL-C; a blood lipid indicator) ratio (MHR) is a new prognostic marker for inflammation associated with cardiovascular diseases. MHR has been used in studies of diabetic retinopathy, nonalcoholic hepatitis and atherosclerosis [7–9]. Carcinoembryonic antigen (CEA), carbohydrate antigen 199 (CA199) are tumor markers that are easily measured during clinical work and have high detection efficiency. Therefore, this study aims to explore the predictive value of MHR combined with tumor markers in CRC and to analyze the relationship between these parameters and the clinicopathological characteristics of CRC patients in order to provide insights to the detection and treatment of CRC.

## Patients and methods

### Patients

A total of 202 CRC patients (CRC group) diagnosed by histopathology who were hospitalized in our hospital from March 2018 to June 2022 were enrolled (mean age, 62.09 ± 12.30 years). 201 healthy individuals (mean age, 59.37 ± 7.67 years; control group) who underwent physical examination at our hospital during the same period were also included. This study was approved by the Ethics Committee of The First Affiliated Hospital of Guangxi Medical University (Approval Number: 2022-E366-01). All the participants were orally informed and agreed to participate. There was no significant difference in age between the two groups. Inclusion criteria for the CRC group: (1) CRC confirmed by histopathology; (2) Hematology, blood biochemistry, blood lipid and tumor marker tests before surgery. Exclusion criteria: (1) After undergoing surgery or treatment; (2) other tumors or malignancies; (3) serious infection and chronic disease.

### Methods

Fasting venous blood was collected from all patients, and monocytes were counted using the LH780 analyzer within 2 h. Aspartate aminotransferase, alanine aminotransferase (ALT), and other parameters were measured using a biochemical analyzer, and CEA, CA199 and carbohydrate antigen 125 (CA125) levels were measured using the Roche immunology analyzer. According to the clinicopathological data of CRC patients, the clinical stage of each patient (stage I to IV) was determined based on the 8th edition of the TNM Classification of Malignant Tumors published by the American Joint Commission on Cancer (AJCC).

### Statistical analysis

Data were analyzed using SPSS 20.0 (IBM Corp, Armonk, NY). Count data were expressed as frequency and percentage, and measured data were expressed as mean ± standard (X ± SD) or mean (quartile). Data were compared between groups using the independent sample T-test or Mann–Whitney U-test, and correlation was analyzed using Spearman correlation analysis. Risk factors for CRC were identified by multivariate logistic regression. A *P* < 0.05 was considered statistically significant.

## Results

### Pathological characteristics of CRC patients

As shown in Table 1, 133 of the 202 CRC were males (65.80%), with a mean age of 62.09 ± 12.30 years. There were 40(19.80%) and 52(25.74%) patients with smoking and drinking history, respectively. Moreover, 34(16.80%), 75(37.10%), 71(35.10%) and 22(10.90%) patients were of pathological stage I, II, III and IV CRC, respectively. A total of 100 (49.50%) patients had lymph node metastasis, 117(57.80%) had rectal tumors, and 85(42.10%) had colon tumors. The tumor size was < 5 cm in 116 cases (57.40%). In addition, 34(16.80%) and 12(5.90%) patients had well-differentiated and poorly differentiated tumors, respectively. A total of 62(30.70%) and 46(22.80%) patients had mass tumors and ulcerating tumors, respectively. 167(82.7%) patients were tested positive for the occult blood test.

**Table 1:** Pathological characteristics of 202 CRC patients

Indicators	Value
Sex, Male, n (%)	133 (65.80)
Age, Male, M ± SD	62.09 ± 12.30
Smoking, n (%)	
Yes	40 (19.80)
No	162 (80.20)
Drinking, n (%)	
Yes	52 (25.74)
No	150 (74.26)
Tumor stage, n (%)	
I	34 (16.80)
II	75 (37.10)
III	71 (35.10)
IV	22 (10.90)
Lymph node metastasis, n (%)	
No	102 (50.50)
Yes	100 (49.50)
Tumor location, n (%)	
Rectum	117 (57.80)
Colon	85 (42.10)
Tumor size, n (%)	
< 5	116 (57.40)
≥ 5	86 (42.60)
Tumor differentiation, n (%)	
Senior	34 (16.80)
Middle +	156 (77.20)
low	12 (5.90)
Tumor type, n (%)	
Mass	62 (30.70)
Infiltration	94 (46.50)
Ulcers	46 (22.80)
Occult blood test, n (%)	
Negative	35 (17.30)
Positive	167 (82.7)

### Comparison of MHR, hematology parameters and tumor markers between CRC and controls

**Fig. 1:**
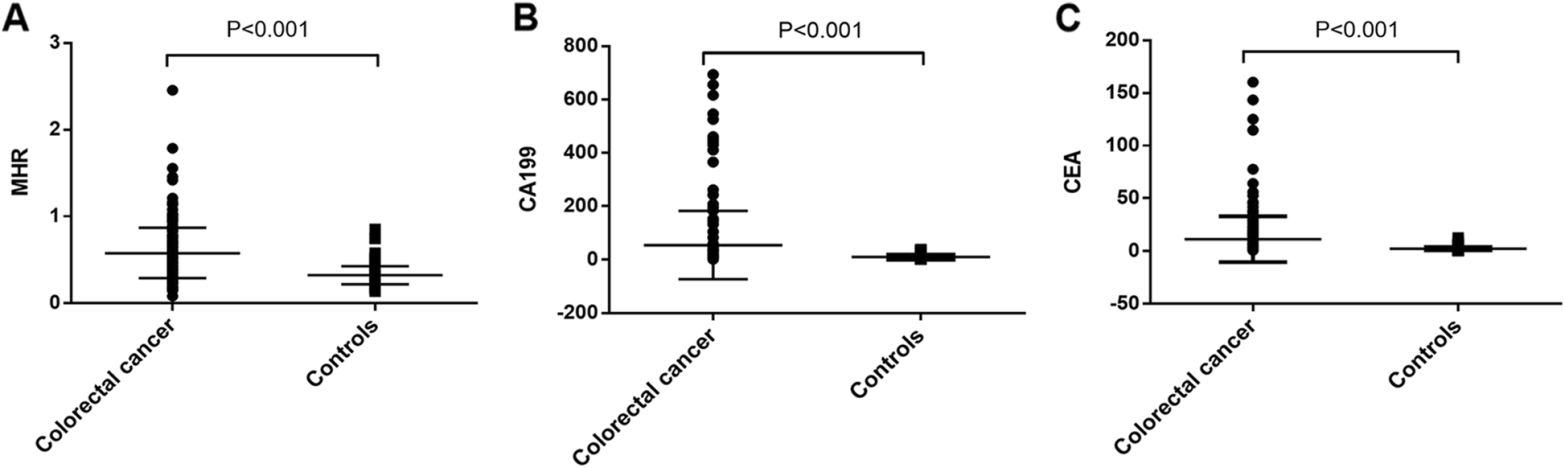
Differences in MHR (**A**), CA199 (**B**), and CEA (**C**) between CRC patients and healthy controls. CA199, carbohydrate antigen 199; CEA, carcinoembryonic antigen; MHR, monocytes to high-density lipoprotein cholesterol ratio

The ALT (*P* = 0.014), total protein (*P* < 0.001), total cholesterol (*P* < 0.001), Triglyceride (*P* < 0.001), M (*P* < 0.001), HDL-C (*P* < 0.001), MHR (*P* < 0.001), CEA (*P* < 0.001), CA199 (*P* < 0.001) levels were significantly different between CRC patients and healthy controls (Table 2). HDL-C was significantly lower in CRC patients than in healthy controls. However, MHR, CEA, and CA199 were significantly higher in CRC patients than in healthy controls (Fig. 1).

**Table 2:** Comparison of MHR, hematology parameters and tumor markers between CRC patients and healthy controls

Indicators	Colorectal cancer	Controls	F	*P*
N	202	201		
Age	60.65 ± 12.23	59.37 ± 7.67	45.57	0.210
ALT	20.20 ± 15.47	23.72 ± 12.98	0.23	0.014
AST	26.83 ± 15.24	24.86 ± 8.12	10.12	0.106
Total protein	68.22 ± 8.41	72.87 ± 4.88	29.14	< 0.001
Total cholesterol	4.81 ± 1.04	4.44 ± 0.69	1053	< 0.001
Triglyceride	1.56 ± 1.25	1.13 ± 0.36	44.53	< 0.001
Monocyte count	0.64 ± 0.30	0.42 ± 0.10	28.50	< 0.001
HDL-C	1.17 ± 0.31	1.32 ± 0.20	29.87	< 0.001
LDL-C	3.01 ± 0.86	2.91 ± 0.17	147.74	0.081
CA153	10.79 ± 6.64	11.08 ± 5.77	43.58	0.637
MHR	0.58 ± 0.29	0.33 ± 0.10	61.48	< 0.001
CEA	3.78 (0.50–160.25)	2.12 (0.50–12.39)	65.27	< 0.001
CA199	10.85 (0.00–693.55)	6.60 (2.00–38.46)	77.29	< 0.001
CA125	10.05 (2.90–203.50)	10.50 (2.50–41.50)	43.58	0.502

The *P* value was calculated by Independent-Samples T-test/ Mann–Whitney U-test

*ALT* alanine aminotransferase, *AST* aspartate aminotransferase, *CA125* carbohydrate antigen 125, *CA153* carbohydrate antigen 153, *CA199* carbohydrate antigen 199, *CEA* carcinoembryonic antigen, *HDL-C* high-density lipoprotein cholesterol, *LDL-C* low-density lipoprotein cholesterol, *MHR* monocytes to high-density lipoprotein cholesterol ratio

### Analysis of the difference between MHR and tumor markers and pathological features of colorectal cancer

**Fig. 2:**
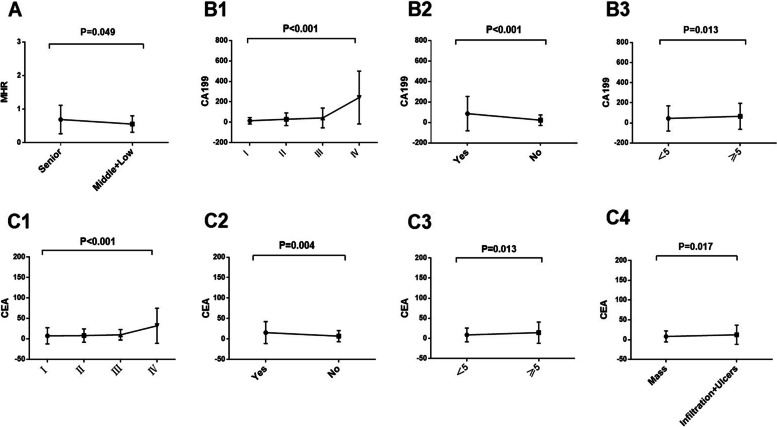
Differences between MHR (**A**), CA199 (**B**), CEA (**C**) and clinicopathological features of CRC. **A**, MHR Vs. Tumor differentiation. **B1**, CA199 Vs. Tumor stage; **B2**, CA199, Vs. Lymph node metastasis; **B3**, CA199 Vs. Tumor size. **C1**, CEA Vs. Tumor stage; **C2**, CEA Vs. Lymph node metastasis; **C3**, CEA Vs. Tumor size. **C4**, CEA Vs. Tumor type. CA199, carbohydrate antigen 199; CEA, carcinoembryonic antigen; MHR, monocytes to high-density lipoprotein cholesterol ratio

As shown in Table 3, there was significant difference between MHR and differentiation degree in 202 CRC patients (*P* = 0.049). CEA was significantly different from tumor stage (*P* < 0.001), lymph node metastasis (*P* = 0.004), tumor size (*P* = 0.013) and tumor type (*P* = 0.017). Meanwhile, there were significant differences between CA199 and tumor stage (*P* = 0.001), lymph node metastasis (*P* < 0.001), and tumor size (*P* = 0.013). Of note, MHR of CRC patients increased with the low degree of tumor differentiation (Fig. 2A). CA199 (Fig. 2B) and CEA (Fig. 2C) levels in CRC patients increased with the increase of late stage, lymph node metastasis and tumor size ≥ 5 cm. Moreover, the CEA levels of CRC patients with ulcerative and infiltrative tumor types are higher than those with mass types (Fig. 2C).

**Table 3:** Relationship among MHR, tumor markers and pathological features of CRC

Indicators	MHR	*P*	CEA	*P*	CA199	*P*
Tumor stage						
I	0.60 (0.22–3.12)	0.941	2.68 (0.55–114.6)	< 0.001	6.45 (2.00–183.31)	0.001
II	0.29 (0.06–1.68)		3.39 (0.5–125.08)		12.75 (0.00–430.00)	
III	0.62 (0.22–1.09)		4.43 (0.75–64.07)		9.80 (0.03–460.00)	
IV	0.59 (0.18–2.25)		18.28 (1.22–160.25)		143.19 (0.20–693.55)	
Lymph node metastasis						
Absence	0.53 (0.08–2.46)	0.593	3.03 (0.50–114.60)	0.004	9.73 (0.00–430.00)	< 0.001
Presence	0.52 (0.16–1.79)		5.68 (0.75–160.25)		11.27 (0.03–693.55)	
Tumor location						
Rectum	0.55 (0.08–1.76)	0.166	3.73 (0.50–143.46)	0.890	11.6 (0.00–693.55)	0.233
Colon	0.50 (0.15–2.46)		3.79 (0.91–160.25)		9.49 (2.00–655.22)	
Tumor size						
< 5 cm	0.52 (0.08–2.46)	0.850	3.33 (0.55–143.46)	0.013	8.76 (0.00–693.55)	0.013
≥ 5 cm	0.54 (0.18–1.46)		4.77 (0.50–160.25)		17.13 (0.03–525.72)	
Tumor differentiation						
Senior	0.51 (0.08–1.79)	0.049	3.36 (0.55–143.46)	0.149	9.28 (2.00–411.51)	0.368
Middle + Low	0.57 (0.18–2.46)		3.92 (0.50–160.25)		11.02 (0.00–693.55)	
Tumor type						
Mass	0.50 (0.18–2.46)	0.407	3.24 (0.75–77.70)	0.017	11.51 (2.00–161.30)	0.685
Infiltration + Ulcers	0.54 (0.08–1.56)		4.12 (0.50–160.25)		10.33 (0.00–693.55)	
Occult blood test						
Negative	0.54 (0.20–1.56)	0.683	3.73 (0.55–77.7)	0.811	9.88 (2.00–525.72)	0.651
Positive	0.53 (0.08–2.46)		3.79 (0.50–160.25)		10.93 (0.00–693.55)	

The *P* value was calculated by Kruskal–Wallis test/Mann–Whitney U test

*CA199* carbohydrate antigen 199, *CEA* carcinoembryonic antigen, *MHR* monocytes to high-density lipoprotein cholesterol ratio

### Correlation between various indexes and pathological characteristics in the CRC group

**Fig. 3:**
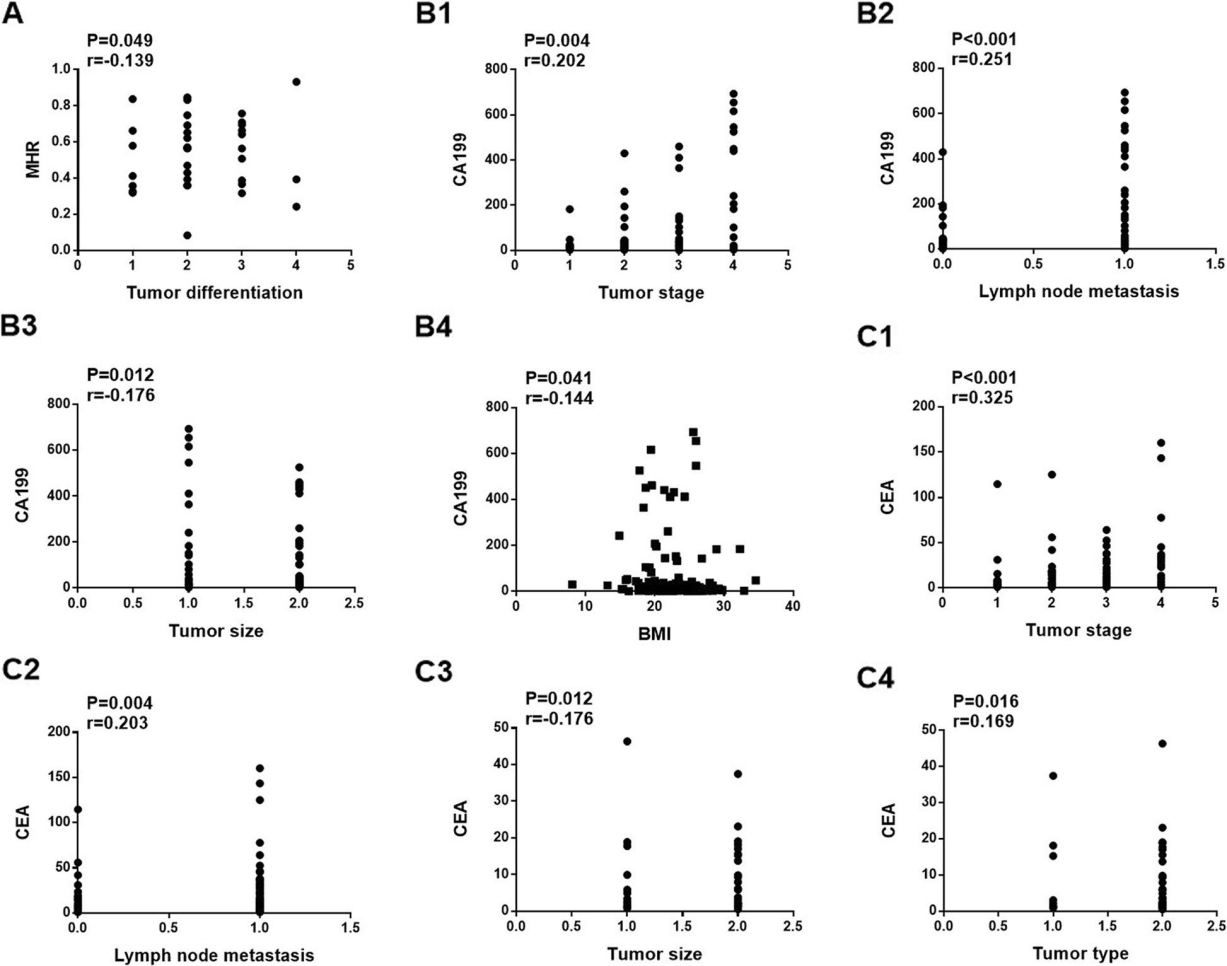
Correlation analysis of MHR (**A**), CA199 (**B**), and CEA (**C**) with clinicopathological features of CRC. **A**, MHR Vs. Tumor differentiation. **B1**, CA199 Vs. Tumor stage; **B2**, CA199 Vs. Lymph node metastasis; **B3**, CA199 Vs. Tumor size; **B4**, CA199 Vs. BMI. **C1**, CEA Vs. Tumor stage; **C2**, CEA Vs. Lymph node metastasis; **C3**, CEA Vs. Tumor size; **C4**, CEA Vs. Tumor type. CA199, carbohydrate antigen 199; CEA, carcinoembryonic antigen; MHR, monocytes to high-density lipoprotein cholesterol ratio

As shown in Table 4, MHR level was positively correlated with the degree of tumor differentiation (*P/r* = 0.049/0.139, Fig. 3A). CA199 (Fig. 3B) was positively correlated with tumor stage (*P/r* = 0.004/0.202), lymph node metastasis (*P/r* =  < 0.001/0.251), tumor size (*P/r* = 0.012/0.176), and negatively correlated with body mass index (*P/r* = 0.041/-0.144). Similarly, CEA (Fig. 3C) was positively correlated with tumor differentiation (*P/r* =  < 0.001/0.325), lymph node metastasis (*P/r* = 0.004/0.203), tumor size (*P/r* = 0.012/0.176), and tumor type (*P/r* = 0.016/0.169) in CRC patients.

**Table 4:** Correlation between various indexes and pathological characteristics of CRC patients

Indicators	MHR	CEA	CA199
	*P*/r	*P*/r	*P*/r
Tumor stage	0.824/0.016	< 0.001/0.325	0.004/0.202
Lymph node metastasis	0.593/-0.038	0.004/0.203	< 0.001/0.251
Tumor location	0.166/-0.098	0.891/-0.010	0.234/-0.084
Tumor size	0.851/-0.013	0.012/0.176	0.012/0.176
Tumor differentiation	0.049/0.139	0.150/0.102	0.369/-0.045
Tumor type	0.409/0.058	0.016/0.169	0.686/-0.029
Occult blood test	0.684/-0.029	0.812/0.017	0.652/0.032
Body mass index	0.107/0.114	0.296/-0.074	0.041/-0.144

The *P* value was calculated by Spearman correlation analysis

*CA199* carbohydrate antigen 199, *CEA* carcinoembryonic antigen, *MHR* monocytes to high-density lipoprotein cholesterol ratio

### CRC risk factors

As shown in Table 5, high levels of MHR (*P* < 0.001) CA199 (*P* = 0.040) and CEA (*P* < 0.001) were independent risk factors for CRC. Especially, individuals with a high MHR level were 14.79 times more likely to suffer from CRC than those with a low MHR level.

**Table 5:** Univariate and multivariate logistic regression analyses of risk factors

	Univariate analysis					Multivariate analysis		
Indicators	B	Exp(B)	*P*	95%Cl	B	Exp(B)	*P*	95%Cl
MHR	2.760	15.81	< .001	9.71–25.73	2.69	14.79	< .001	8.57–25.55
CEA	0.32	1.38	< .001	1.24–1.53	0.25	1.29	< .001	1.17–1.54
CA199	0.03	1.026	0.001	1.01–1.04	0.03	1.03	0.040	1.00–1.05

*CA199* carbohydrate antigen 199, *CEA* carcinoembryonic antigen, *MHR* monocytes to high-density lipoprotein cholesterol ratio

### Comparison of 202 CRC patient’s clinicopathological features stratified by MHR, CEA, CA199

As shown in Table 6, the cutoff values for MHR, CEA, and CA199 are 0.387, 3.61, and 12.95, respectively. According to their cutoff values, the clinical and pathological characteristics of 202 CRC patients were stratified and compared. The results showed that low and high levels of CEA were most closely related to the staging, lymph node metastasis, tumor size, and other factors of CRC patients (*P* < 0.05).

**Table 6:** Comparison of 202 CRC patient’s clinicopathological features stratified by MHR, CEA, CA199

Indicators	MHR		*P*	CEA		*P*	CA199		*P*
	≤ 0.387	> 0.387		≤ 3.61	> 3.61		≤ 12.95	> 12.95	
Tumor stage									
I + II	21	88	0.728	61	47	0.004	64	45	0.669
III + IV	20	73		33	60		51	42	
Lymph node metastasis									
Absence	20	82	0.862	59	43	0.002	60	42	0.670
Presence	21	79		36	64		55	45	
Tumor size									
< 5 cm	21	95	0.382	62	54	0.046	75	41	0.014
≥ 5 cm	20	66		33	53		40	46	
Tumor differentiation									
Senior	4	30	0.243	19	15	0.266	20	14	0.851
Middle + Low	37	131		76	92		95	73	
Tumor type									
Mass	15	47	0.448	36	26	0.047	32	30	0.356
Infiltration + Ulcers	26	114		59	81		83	57	

The *P* value was calculated by chi square test. The numbers in the table represent the number of samples

*CA199* carbohydrate antigen 199, *CEA* carcinoembryonic antigen, *MHR* monocytes to high-density lipoprotein cholesterol ratio

### Diagnostic efficacy of MHR and tumor markers in CRC

**Fig. 4:**
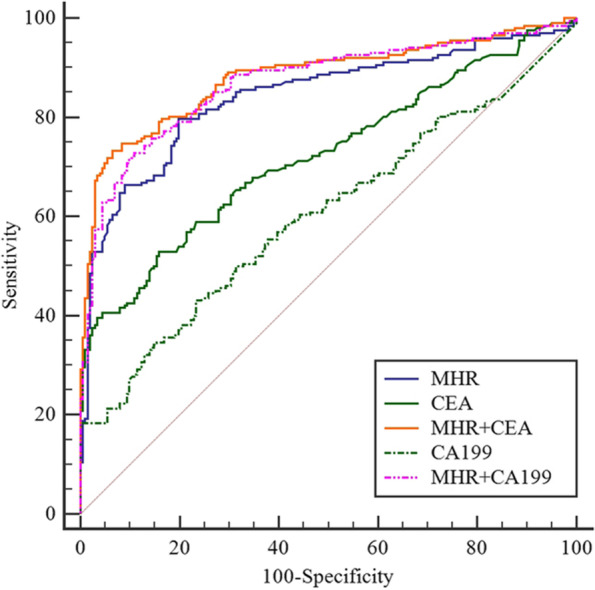
Diagnostic efficiency (ROC curve). CA199, carbohydrate antigen 199; CEA, carcinoembryonic antigen; MHR, monocytes to high-density lipoprotein cholesterol ratio

The area under ROC curve (AUC) of MHR, CEA, CA199 for the diagnosis of CRC was 0.842, 0.723, 0.604, respectively. However, the AUC of MHR combined with CEA, and CA199 for the diagnosis of CRC was 0.882, and 0.869, respectively, which were higher than those of CEA, and CA199 alone (Table 7, Fig. 4).

**Table 7:** Diagnostic efficacy of MHR combined with tumor markers for CRC

Indicators	AUC (95%Cl)	Youden	SENS	SPE	z-statistic	*P*
MHR	0.842 (0.802–0.876)	0.60	79.70	80.10	16.67	< 0.001
CEA	0.723 (0.676–0.766)	0.37	52.97	84.08	8.78	< 0.001
CA199	0.604 (0.554–0.652)	0.20	43.07	76.62	3.665	< 0.001
MHR + CEA	0.882 (0.846–0.944)	0.67	73.27	93.53	21.49	< 0.001
MHR + CA199	0.869 (0.832–0.901)	0.87	71.78	90.05	19.97	< 0.001

*CA199* carbohydrate antigen 199, *CEA* carcinoembryonic antigen, *MHR* monocytes to high-density lipoprotein cholesterol ratio, *SENS* Sensitivity; SPE, Specificity

## Discussion

Malignant tumors are associated with the occurrence and development of chronic inflammation. The basic pathological processes of chronic inflammation include endothelial damage, lipid deposition, and oxidative stress [10]. MHR, which relies on a combination of M count and HDL-C, has recently become a new and convenient inflammatory marker. In our study, the hematological data of the 202 CRC patients and 201 healthy controls showed that M count and MHR were higher whereas HDL-C was lower in CRC patients than in the healthy controls. This was consistent with the higher MHR level observed in patients with papillary thyroid cancer and diabetic retinopathy [7, 11]. In addition, a high MHR level has also been used to evaluate disease prognosis. For example, MHR is an independent predictor for long-term hospitalization and mortality in patients with acute coronary syndrome or a post-PCI status, as well as for adverse prognosis related to heart disease [12]. Furthermore, recent studies have found that increased MHR is a new predictor for metabolic disorders, including metabolic syndrome and polycystic ovary syndrome [13, 14].

MHR can reflect both inflammation and lipid accumulation since monocytes are an inflammatory marker and HDL-C is a blood lipid indicator. Hyperlipidemia has been shown to impair the functions of the arterial intima. Low-density lipoprotein (LDL) enters the intima, followed by oxidative modification, which damages the intima. Damaged vascular endothelial cells express adhesion molecules which allow monocytes to bind and migrate to the lower endothelium and mature into macrophages. Oxidized LDL-C promotes the release of pro-inflammatory cytokines and induces chronic inflammation, thereby facilitating the occurrence and development of CRC. HDL is involved in cholesterol reverse transport and has antioxidative and anti-inflammatory effects [15]. Low Cholesterol efflux mediated by low or damaged HDL can lead to the increased monocytes, thus promoting the progression of chronic inflammation [16]. HDL can inhibit the expression of tissue factor in monocytes by preventing the activation of p38c and phosphoinositol 3 kinase [17], downregulate F-actin to prevent monocytes from gathering and adhering to the surface of the vascular endothelium, regulate the activation, proliferation and differentiation of monocytes [18], and thereby effectively inhibit the progression of oxidative stress and inflammatory response [19]. Since monocytes and HDL play significant roles in promoting or inhibiting inflammation and antioxidation [20], respectively, the ratio of these two indicators (MHR) can be used to assess the inflammatory state of the patients and the occurrence and development of chronic inflammation-related diseases, which is more advantageous than a single indicator. Therefore, a high plasma MHR level can serve as an indicator for tumor-related inflammation.

When the relationship between MHR level and the clinicopathological characteristics of CRC patients was analyzed, it was found that MHR level was positively correlated the degree of tumor differentiation in CRC patients. The degree of tumor differentiation is defined as the closeness between tumor cells and normal cells. The lower the degree of tumor differentiation, the higher the malignancy of the tumor. Tumor tissues are similar to the immature morphology of the source tissue, which are greatly different from the corresponding normal source tissue, and grow faster and metastasize more easily. In this study, the MHR increased as the degree of tumor differentiation decreased, indicating that the tumors in patients with high MHR tend to be poorly differentiated, and the degree of malignancy is higher in patients with high MHR than in those with low MHR. Therefore, a continuous increase of MHR serves as a promising marker for CRC progression.

It is well known that serum tumor markers are closely associated with tumor occurrence and development, including CEA, CA199. CEA is a glycoprotein produced by CRC tissues which can be used to assess the existence, development and prognosis of various tumors clinically. CA199 has been used for the diagnosis of adenocarcinoma since its discovery in 1979, and is expressed in various malignant tumors. It is commonly used for disease detection and efficacy evaluation, and is particularly detected in CRC. Some studies have shown that simultaneous detection of various tumor markers can improve the detection rate of malignant tumors [21, 22]. In this study, the level of CEA, CA199 were higher in CRC patients than in healthy controls, which is similar to the findings of Bjorkman et al. [23], Lakemeyer et al. [24] and Luo et al. [25].

TNM staging developed by the AJCC is the most widely used CRC staging system and has demonstrated remarkable value in early evaluation, surgical selection, treatment scheme selection and prognosis evaluation [26]. Therefore, we used the TNM system to classify the pathological characteristics of CRC patients, and it was found that CA199 and CEA were positively correlated with cancer stage, lymph node metastasis, tumor size. Moreover, CEA was also significantly correlated with tumor type. Our results showed that CEA and CA199 levels in CRC patients increased with increased stage, lymph node metastasis and tumor size ≥ 5 cm, which suggested that CEA and CA199 are closely associated with the clinicopathological characteristics of CRC. In particular, high levels of CEA and CA199 can guide the clinicopathological staging of CRC, which is similar to the results of Björkman K et al. [23]. It was also found that high levels of CA199 and CEA were closely associated with lymph node metastasis. Consistent with previous studies [27, 28], we found no correlation between CEA and CA199 levels and tumor differentiation. Interestingly, CA199 was also negatively correlated with body mass index, which may be attributed to the more severe the condition of cancer patients, the more pronounced the cachexia of the body.

The AUC of the ROC curves indicated that MHR has the highest diagnostic efficacy for CRC compared with CEA and CA199. However, combination of MHR with CEA and CA199 showed better diagnostic efficacy and was closely associated with the clinical features of CRC.

Several limitations of this study include the retrospective nature of the study, small sample size, and the same region of origin of the patients, which may introduce selection bias in the data. Therefore, a large-cohort multicenter study will be warranted to further confirm these findings.

## Conclusion

In summary, this is the first study to show that a continuous increase of MHR is an independent risk factor for CRC. MHR is a promising new indicator, and its combination with CEA and CA199 has great predictive value for CRC progression.

## Data Availability

The data generated in the present study are included in the figures and/or tables of this article.

## Abbreviations

ALT: Alanine aminotransferase
AST: Aspartate aminotransferase
CA125: Carbohydrate antigen 125
CA153: Carbohydrate antigen 153
CA199: Carbohydrate antigen 199
CEA: Carcinoembryonic antigen
CRC: Colorectal cancer
HDL-C: High-density lipoprotein cholesterol
LDL-C: Low-density lipoprotein cholesterol
MHR: Monocytes to high-density lipoprotein cholesterol ratio
